# Relationship between motor dysfunction, the respiratory muscles and pulmonary function in stroke patients with hemiplegia: a retrospective study

**DOI:** 10.1186/s12877-023-04647-x

**Published:** 2024-01-13

**Authors:** Meng Li, Ying Huang, HaiYun Chen, ShuoShuo Wang, Ying Zhou, Ying Zhang

**Affiliations:** 1 Department of Rehabilitation, Shanghai Xuhui Central Hospital, Shanghai, China; 2 Department of Pneumology, Shanghai Xuhui Central Hospital, Shanghai, China; 3 Department of Geriatrics, Shanghai Xuhui Central Hospital, Shanghai, China

**Keywords:** Hemiplegia, Stroke, Motor function, Respiratory muscles, Pulmonary function

## Abstract

**Background:**

The interaction between motor dysfunction and respiratory functions in stroke patients with hemiplegia are not fully understood, particularly with regard to the relationship between changes in trunk control, balance, and daily activities, and changes in respiratory muscle strength and pulmonary volume. Investigating this relationship will facilitate the optimization of stroke rehabilitation strategies.

**Methods:**

Clinical history data were collected from 134 patients to analyze the relationship between motor function scales scores and spirometric data. The data from 60 patients’ data were used to evaluate the relationship between motor function scales scores and spirometric data at baseline and after 3-weeks rehabilitation.

**Results:**

(1) Patients with lower scores on Trunk impairment Scale (TIS), Berg Balance Scale (BBS) and Barthel Index (BI) had weaker respiratory muscle strength and pulmonary function. (2) Stroke patients’ BBS and BI scores showed differences between normal and unnormal maximal inspiratory pressure (MIP), but not in TIS. (3) Improvements in motor function led to promotion of enhanced respiratory function. Patient exhibited less MIP improvement at the severe level of TIS and BBS.

**Conclusions:**

Patients with hemiplegia exhibited diminished respiratory muscle strength and pulmonary function at a more severe motor dysfunction level. Impaired inspiratory muscle strength was associated with reduced balance ability and limitations in activities required for daily living. Enhanced motor function improved respiration and rehabilitation programs should prioritize the activation of diaphragm function to improve overall outcomes.

## Introduction

The movement of torsos in both hemiplegic and non-hemiplegic stroke patients is obviously [1]. Fuglmeyer proposed that respiratory dysfunction was common in stroke patients [2], and further that deep core muscles play a vital role in maintaining trunk posture and respiration [3].

Abnormalities in trunk control, such as reduced abdominal muscle activity and loss of selective trunk activity, results in the loss of flexion, rotation, and lateral flexion movements of the torso, as well as a lack of synchronized activation between the trunk and limb muscles [4]. Trunk dysfunction after stroke also produces impaired balance and physical activities essential for daily living [5]. Hyperactive back extensor muscle activity in patients often leads to abnormal lifting of the ribs and chest, which reduces the range of motion of the diaphragm and affects the activity of abdominal and deep core muscles [6], ultimately having an impact on lung capacity [7]. Research has shown that forced vital capacity and maximum expiratory and inspiratory pressures are increased in stroke patients with hemiplegia after specialized trunk control training [8].

In turn, weakened respiratory muscles made it difficult for those patients to maintain proper breathing pattern, lead to a deterioration in trunk function, balance, and daily activities [9]. Early stroke patients often experience a marked reduction in respiratory muscle strength on both the hemiplegic and non-hemiplegic side [10], which was be worsened by prolonged confinement to bed. This caused reduced cough ability, difficulty in sputum excretion, and eventually in the worst cases to pneumonia [11]. Studies have shown that respiratory muscle training improves maximal inspiratory pressure, inspiratory muscle endurance and peak expiratory flow in the short term, thereby promoting coughing ability [12] and improving indicators such as the Trunk Impairment Scale (TIS) and Barthel Index (BI) [13]. Research has shown that 6 weeks of respiratory muscle training effectively increases the speed and distance of patients’ centre of pressure shift [14] and 8 weeks of inspiratory training improves the patient’s reflexes and ability to perform dynamic tasks [15], which are required for balance.

Respiratory and motor function training are both important treatments for post-stroke rehabilitation [16, 17]. However, the relationships between trunk capacity, balance, and activities of daily living to the inspiratory, and expiratory muscles, as well as lung volume at different levels of impairment after stroke, remain unclear. Expiratory muscle rehabilitation for stroke patients has not received much attention that it deserves until relatively recently [18]. Indeed, the profession of respiratory therapists was not established in China until 2019, and still at present problems remain, such as insufficient attention to respiratory training and inconsistent operating standards [19]. It is imperative to investigate the impact of routine rehabilitation training on the respiratory function of patients with hemiplegia, as it may contribute to the optimization of post-stroke rehabilitation programs. Therefore, a retrospective study was conducted to explore these aspects in patients with post-stroke hemiplegia. The findings of this study may have significant implications for enhancing the motor performance and respiratory capacity of post-stroke patients.

## Methods

### Study design and patients

This was a retrospective study that received approval from ShangHai Xuhui Central Hospital Ethics Committee (No.2022-043). Due to the nature of a retrospective study, the need for informed consent was waived by ShangHai Xuhui Central Hospital Ethics Committee. Data were de-identified prior to analysis.

Ultimately the clinical history data of 134 patients at hospital admission, including gender, age, height, weight, disease type, duration of onset, motor function assessment results and pulmonary capacity results were collected and analyzed. In addition, 60 of 134 patients’ motor function assessment and pulmonary capacity results at discharge were obtained.

**Fig. 1:**
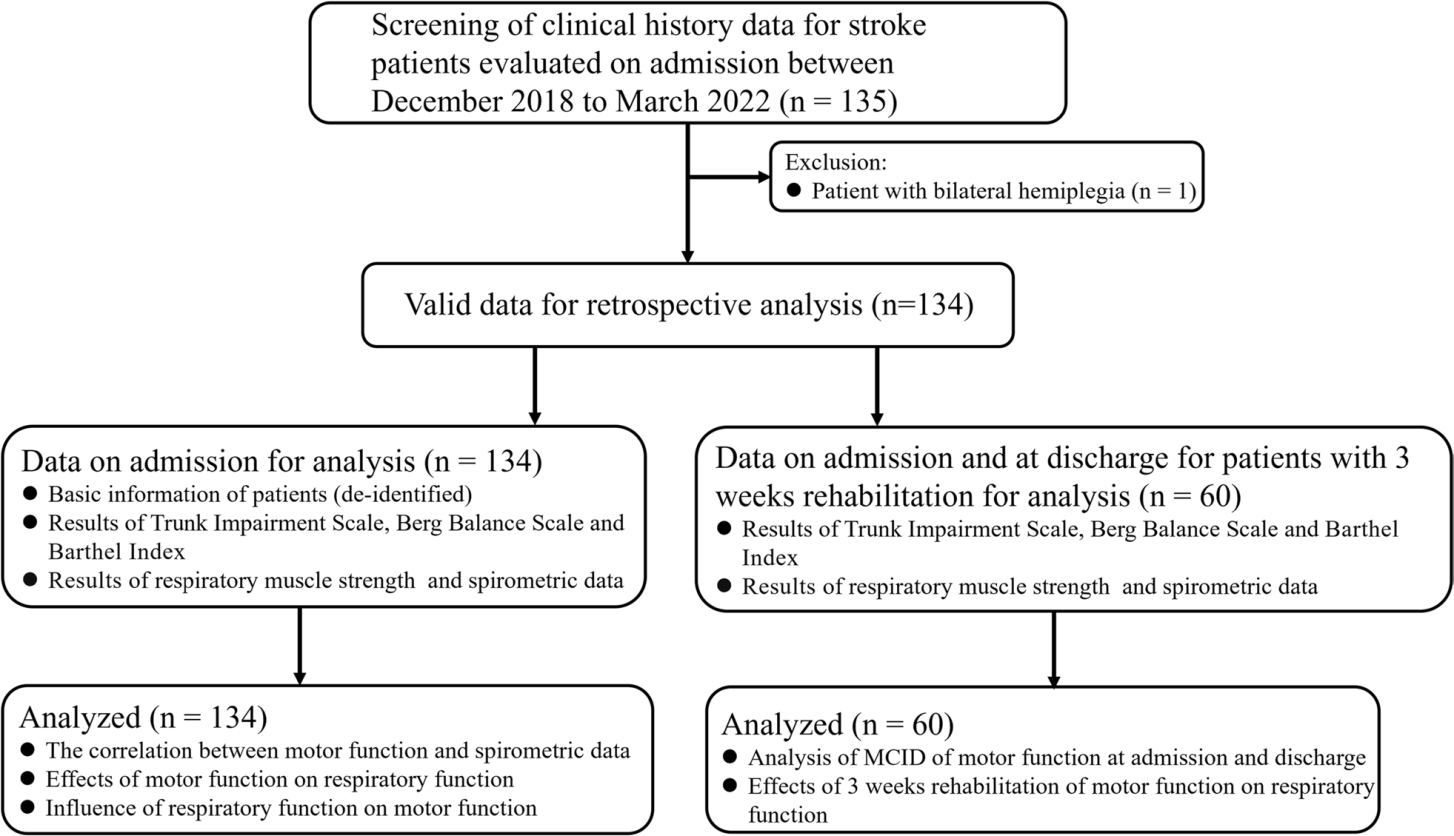
Research flow chart

Through electronic medical records, the data of stroke patients hospitalized in the Rehabilitation Department of Xuhui District Central Hospital between December 2018 and March 2022 were reviewed. Patients had to meet the following criteria to be included in the study: 1) a diagnosis of cerebral infarction or cerebral hemorrhage; 2) met the diagnostic criteria for stroke; 3) hemiplegia patients with a first episode of stroke; 4) aged ≥ 18 years; 5) sitting balance of ≥ grade 1; and 6) had a report of a pulmonary ventilatory function test. Exclusion criteria were: 1) had consciousness disorder and severe cognitive dysfunction; and 2) had acute diseases of the heart, brain, kidney and other organs. The research process is shown schematically in Fig. 1.

### Evaluation of motor functions

Trunk function has been identified as an important early predictor of functional outcomes after stroke. The TIS is a valid tool for examining person with hemiplegia’ trunk control ability, allowing for both qualitative and quantitative assessment of trunk deficits [20]. TIS consists of 3 subscales: namely, static, dynamic sitting balance and trunk coordination in a sitting position, with a total maximum score of 23 [21]. According to previous studies, non-ambulatory patients had a median TIS score of 8 and ambulatory patients had a median score of 14 (11–18) [22]. Although it was not a direct reference basis, we believe it was a relatively appropriate method for our research. We took 8 points and 18 points as two nodes and divided patients into 3 groups based on their TIS scores and classified thus: a patient score of 0–7 points as a severe level, 8–17 points a moderate level and 18–23 points a slight level.

The Berg Balance Scale (BBS) is a comprehensive balance function examination scale, which evaluates the patient’s ability to shift actively their centre of gravity through multiple functional activities, including dynamic and static balance tasks while sitting and standing. It had 14 item list with each item consisting of a 5-point ordinal scale ranging from 0 to 4. 0 indicated the lowest level of function and 4 was the highest level. Scores of 0 to 20 suggested that patients had poor balance and needed a wheelchair. Scores of 21–40 indicated that patients had a certain balance ability and could walk with assistance. Scores of 41–56 showed that patients can walk independently [23].

The BI was used to measure the degree of assistance required for patients’ activities involved in daily living, which included 10 personal activities with a total score of 100 points. Guidelines for interpreting BI scores are: scores of 0–20 indicate “total” dependency; 21–60 indicate “severe” dependency; 61–90 indicate “moderate” dependency; and 91–99 indicate “slight” dependency [24].

### Evaluation of spirometry

Spirometric data were evaluated through a portable pulmonary function instrument (Xeek X1, China) with the patients in a sitting position. The measured value of the parameter was expressed as a percentage of the predicted value. The main collected parameters were as follows: 1) maximal inspiratory pressure (MIP), male normal value ≥ 75% (of predicted value), female normal value ≥ 50%; 2) maximal expiratory pressure (MEP), male normal value ≥ 100%, female normal value ≥ 80%; 3) forced vital capacity (FVC): the total volume of air exhaled with maximum strength and fastest speed; 4) forced expiratory volume in 1 s(FEV_1_), evaluated the volume of air exhaled by the patient using the maximum force and the fastest speed in 1 s, which reflected ventilation dysfunction and was dependent on the respiratory muscles and airway status [25]. It is divided into 5 different levels: mild, FEV_1_ over 70%; moderate, FEV_1_ 60–70%; moderately severe, FEV_1_ 50–60%; severe, FEV_1_ 35–50%; very severe, FEV_1_ < 35% [26]. 5) peak expiratory flow (PEF) reflecting the highest flow rate during forced expiration, which is an important index reflecting the strength of the expiratory muscles [27]; and 6) maximal mid-expiratory flow (MMEF) representing the mean expiratory flow rate at which 25%-75% of vital capacity is exhaled with force.

### Statistical analysis

The data were analyzed using SPSS 23 (SPSS Inc., 233S. Wacker Dr., IL). Categorical data are presented as percentages and continuous data as means ± standard deviation (SD) and medians (interquartile ranges).

Spearman’s correlation coefficient was employed to analyze the relationship between values in motor function scales and respiratory indicators. The Wilcoxon signed-rank test was utilized to analyze any differences in spirometric data among individuals with varying degrees of motor dysfunction, as well as the differences in motor function across different spirometric indexes. Additionally, we used the Minimal Clinically Important Difference (MCID) of TIS, BBS and BI to determine if genuine changes in a patient’s function had occurred [28]. We also analyzed the changes in lung function indexes after 3 weeks of physical therapy intervention. Finally, the study investigated the differences in the change values of spirometric indexes among individuals with different levels of motor dysfunction following the 3-week physical therapy intervention. A significance level (*p*) of 0.05 was determined for the statistical comparisons.

## Results

The details of demographic and related indicators are presented in Table 1. The data of 100 males and 34 females were analyzed, the average age was 66.7 ± 10.1 years and the average post-stroke duration was 42.3 ± 34.8 days; 114 (85.1%) patients were diagnosed with ischemic stroke, and 20 (14.9%) with hemorrhagic stroke. Left hemiplegia was 57 with a proportion of 42.5%, while right hemiplegia was 77 with a proportion of 57.5%.

**Table 1:** General characteristics of patients at admission

	**Value at T**_**0**_
General, n (%)	
Male	100 (74.6)
Female	34 (25.3)
Age, years (Mean ± SD)	66.7 ± 10.1
Post-stroke duration, days (Mean ± SD)	42.3 ± 34.8
Type, n (%)	
Ischemic	114 (85.1)
Hemorrhagic	20 (14.9)
Hemiplegic side, n (%)	
Left	57 (42.5)
Right	77 (57.5)
TIS (Points, Mean ± SD)	12.0 ± 4.3
BBS (Points, Mean ± SD)	19.9 ± 16.4
BI (Points, Mean ± SD)	47.5 ± 15.5
MIP (%, Mean ± SD)	36.7 ± 18.8
MEP (%, Mean ± SD)	38.7 ± 22.5
FVC (%, Mean ± SD)	72.9 ± 24.9
FEV_1_ (%, Mean ± SD)	70.2 ± 27.2
PEF (%, Mean ± SD)	38.3 ± 23.8
MMEF (%, Mean ± SD)	57.7 ± 31.8

*T*_*0*_ The time of admission, *TIS* Trunk Impairment Scale, *BBS* Berg balance scale, *BI* Barthel index, *MIP* Maximal inspiratory pressure, *MEP* Maximal expiratory pressure, *FVC* Force vital capacity, *FEV*_*1*_ Forced expiratory volume in one second, *PEF* Peak expiratory flow, *MMEF* Maximal mid expiratory flow

### Correlation between motor function and spirometric data

**Fig. 2:**
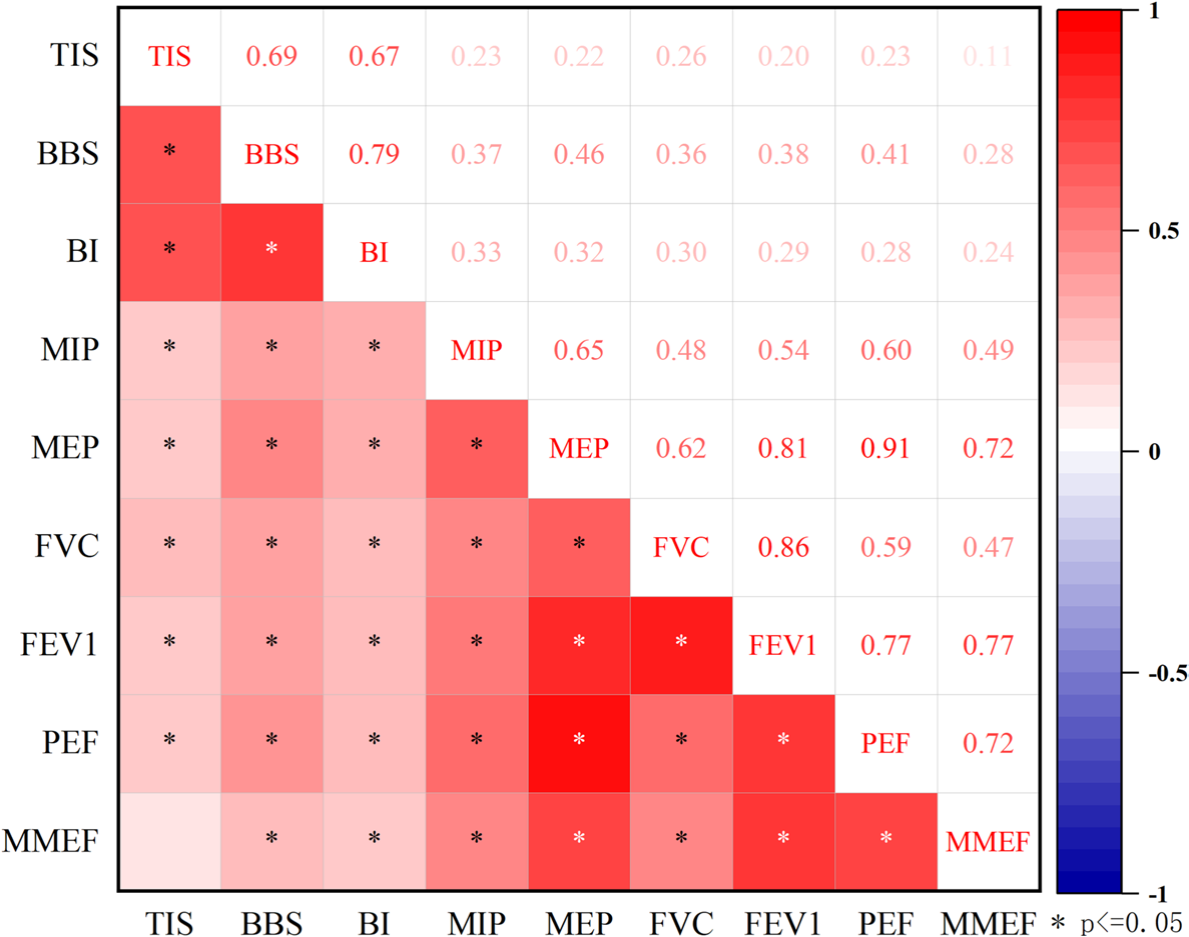
Spearman’s correlation between motor function and spirometric data

The results of Spearman’s correlation coefficient analysis are presented in Fig. 2. TIS was shown to have slight positive correlations with MIP, MEP, FVC, FEV_1_ and PEF.

BBS showed moderate positive correlations with MIP (*r* = 0.37, *P* < 0.001, PEF (*r* = 0.40, *P* < 0.001), and slight positive correlations with MEP, FVC, FEV_1_ and MMEF. BI indicated moderate positive correlations with MIP (*r* = 0.33, *P* < 0.001), MEP (*r* = 0.32, *P* < 0.001), FVC (*r* = 0.3, *P* < 0.001), and slight positive correlations with MEP, FVC, FEV_1_ and MMEF. FEV_1_ and PEF and MMEF.

### Respiratory functions at multiple motor dysfunction levels

**Fig. 3:**
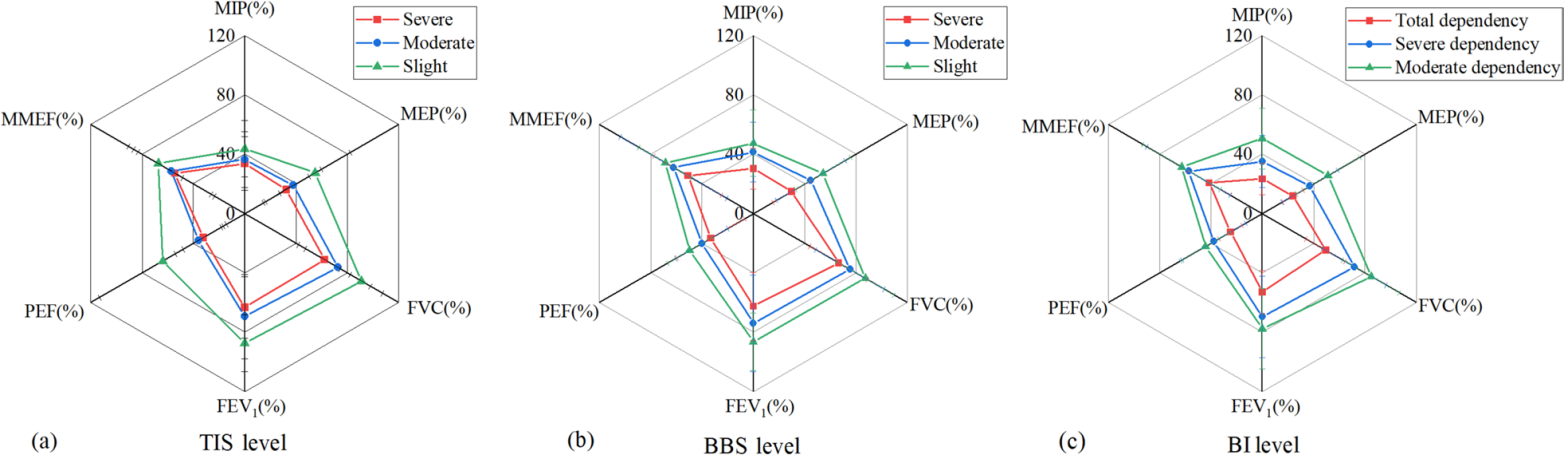
**a** Spirometric data at three levels of TIS (**a**), BBS (**b**) and BI (**c**)

TIS determined a patient’s trunk control ability at 3 levels, namely severe, moderate and slight. The MIP values were 29.0 (20.3–44.0), 33.7 (22.4–45.3) and 38.4 (28.1–59.4) for the 3 levels, respectively with statistical significance (*P* = 0.029). The MEP was 29.0 (19.4–38.0), 33.3 (23.1–46.8) and 45.8 (29.4–80.5) for the 3 levels, respectively with significant differences (*P* = 0.002). Significant effects were also found with FVC (*P* = 0.014) and FEV_1_ (*P* = 0.011) for the 3 levels of TIS (Table 2, Fig. 3a). The findings indicated that different trunk control abilities showed differences in these respiratory indicators. Thus, increasing trunk control ability will clearly help in improving these respiratory indicators.

**Table 2:** Spirometric data (Median (Q1-Q3)) at three levels of trunk Impairment, Berg Balance Scale and Barthel Index

	**Trunk Impairment Scale (TIS)**				**Berg Balance Scale (BBS)**				**Barthel Index (BI)**			
	**Severe (*N* = 20)**	**Moderate (*N* = 101)**	**Slight (*N* = 13)**	*P*	**Severe (*N* = 68)**	**Moderate (*N* = 43)**	**Slight (*N* = 23)**	*P*	**Total (*N* = 6)**	**Severe (*N* = 108)**	**Moderate (*N* = 20)**	*P*
**MIP (%)**	29.0 (20.3–44.0)	33.7 (22.4–45.3)	38.4 (28.1–59.4)	0.029	29.1 (18.5–38.0)	36.2 (28.4–47.3)	43.9 (33.2–64.6)	< 0.001	20.5 (17.2–28.5)	32.2 (22.3–44.7)	46.8 (32.8–66.7)	< 0.001
**MEP (%)**	29.0 (19.4–38.0)	33.3 (23.1–46.8)	45.8 (29.4–80.5)	0.002	28.4 (19.2–39.2)	37.2 (28.9–59.6)	54.6 (36.1–71.9)	< 0.001	26 (22.6–29)	31.8 (23.6–43.8)	56.3 (34.3–71.9)	0.008
**FVC (%)**	63.3 (47.2–78.3)	71.1 (54.7–89.1)	94.7 (78.5–103.8)	0.014	65.8 (48.1–86.6)	71.1 (57.7–86.2)	94.6 (77.9–103.8)	0.002	55.3 (48.4–58.8)	70.6 (54.7–88.3)	85.3 (69.5–101.4)	0.005
**FEV**_**1**_** (%)**	59.7 (48.9–77.9)	67.4 (49.2–85.2)	88.7 (71.7–100.4)	0.011	61.9 (43.9–79.2)	67.4 (57.3–84.4)	89.2 (76.5–99.1)	< 0.001	57.3 (52.3–60.9)	69.7 (50.8–85)	83.8 (54–99.1)	0.051
**PEF (%)**	31.9 (19.8–38.2)	33.6 (23.3–47.6)	45.9 (40.6–75.3)	0.154	30.2 (19.6–41.1)	37.7 (27.7–52.4)	48.9 (35.3–64.1)	< 0.001	27.1 (22.8–31.7)	33.6 (24.9–46)	48.2 (31.1–58)	0.04
**MMEF (%)**	46.6 (33.1–70.6)	52.4 (35.8–71.0)	68.7 (53.6–84.3)	0.254	47 (34–67.1)	52.4 (35.3–73.5)	64.6 (54.6–82.4)	0.01	36.5 (34.7–46.5)	52.6 (36–71.8)	59.2 (43.3–88.3)	0.223

BBS evaluates a patient’s balance ability at 3 levels, namely severe, moderate, and slight. Table 2 and Fig. 3b illustrate the spirometric data at 3 different balance ability levels. MIP values were 29.1 (18.5–38.0), 36.2 (28.4–47.3) and 43.9 (33.2–64.6), respectively for the 3 levels with statistical significance reached (*P* < 0.001). The MEP was 28.4 (19.2–39.2), 37.2 (28.9–59.6) and 54.6 (36.1–71.9), respectively for the 3 levels all with significant differences (*P* < 0.001). Significant differences were also found in FVC (*P* = 0.002), FEV_1_ (*P* < 0.001), PEF (*P* < 0.001), and MMEF (*P* = 0.01) at the 3 BBS levels. The results indicated that different balance abilities reflect differences in these respiratory indicators, thus increasing balance ability should help to improve respiratory indicators.

BI assessed the patient’s dependency on daily living activities, which included 5 levels. This study included 3 levels, namely total, severe and moderate dependency due to the highest BI scores being ≤ 90 points. The results indicated that patients’ respiratory muscle strength decreased with increasing dependency on living activities. MIP values were 20.5 (17.2–28.5), 32.2 (22.3–44.7) and 46.8 (32.8–66.7) for 3 levels with significant differences (*P* < 0.001). MEP values were 26 (22.6–29), 31.8 (23.6–43.8) and 56.3 (34.3–71.9), respectively for 3 levels with significant differences (*P* = 0.008). Significant differences were associated with FVC (*P* = 0.005) and PEF (*P* = 0.04) on three BI levels (Table 2, Fig. 3c). The results indicated that various daily living abilities exhibited differences in these respiratory indicators, thus increasing daily living abilities should help to facilitate these respiratory indicators.

### Motor dysfunction at 2 levels of respiratory muscle strength

Between normal and abnormal MIP levels, TIS was 13 (10–15) and 15 (13–16) points, with no significant differences (*P* = 0.099), whereas BBS were 17 (3–32.5) and 33 (29–42), with significant differences (*P* = 0.02), and BI were 50 (35–55) and 62.5 (50–70), also with significant differences (*p* = 0.022) (Table 3).

**Table 3:** TIS, BBS and BI values (Median (Q1-Q3)) with normal and abnormal MIP and MEP level and five different FEV_1_ levels

	**Maximal inspiratory pressure (MIP)**			**Maximal expiratory pressure (MEP)**			**Forced expiratory volume in 1 s (FEV**_**1**_**)**					
	**Abnormal (*N* = 124)**	**Normal (*N* = 10)**	***p***	**Abnormal (*N* = 131)**	**Normal (*N* = 3)**	***p***	**Mild (*N* = 67)**	**Moderate (*N* = 19)**	**Moderately Severe (*N* = 18)**	**Severe (*N* = 18)**	**Very Severe (*N* = 12)**	***p***
TIS (Point)	13 (10–15)	15 (13–16)	0.099	13 (10–15)	13 (7.5–15)	0.944	13 (11–15)	14 (11–15)	14 (6–15)	11.5 (8–14)	11 (10–14)	0.330
BBS (Point)	17 (3–32.5)	33 (29–42)	0.02	20 (3–35)	31 (17.5–33.5)	0.597	25 (9–42)	21 (2.5–28)	22 (1–35)	3 (1–16)	15.5 (1–23)	0.004
BI (Point)	50 (35–55)	62.5 (50–70)	0.022	50 (35–60)	55 (42.5–57.5)	0.876	50 (40–60)	45 (35–55)	50 (30–60)	35 (30–55)	45 (35–60)	0.044

Between normal and abnormal MEP levels, TIS were 13 (10–15) and 13 (7.5–15) points, with no significant differences (*P* = 0.944), BBS were 20 (3–35) and 31 (17.5–3.5), with no significant differences (*P* = 0.597), and BI were 50 (35–60) and 55 (42.5–57.5), again with no significant differences (*P* = 0.876) (Table 3).

###  Motor dysfunction at 5 pulmonary ventilation dysfunction levels (FEV_1_)

The FEV_1_ was divided into five different levels, including mild: FEV_1_ over 70%; moderate: FEV_1_ 60–70%; moderately severe: FEV_1_ 50–60%; severe: FEV_1_ 35–50%; very severe: FEV_1_ < 35%. Table 3 shows no significant difference in TIS (*P* = 0.33), however, there were significant differences in BBS (*P* = 0.004) and BI (*P* = 0.044) in 5 different ventilatory dysfunction levels. The patients with higher FEV_1_ values tended to have better balance ability and independence in daily activities.

### The progress of motor function brought changes in spirometric data after 3-weeks rehabilitation

Regular rehabilitation training used the Bobath technique to inhibit abnormal posture and movement patterns, to induced postural reflex and balance reaction, and to promote the formation of normal movement patterns. It included joint movement training, supine turnover and bridge training, transfer of the torso while sitting and standing, and ball activities to build muscle strength and endurance, improve static and dynamic balance, and enhance walking ability. All patients did not receive specific breathing training.

The correlation coefficients between the two assessment results of TIS, BBS and BI were 0.840, 0.947 and 0.956. MCID were 1.5, 3.4 and 4.3 points respectively (Table 4). MCID determined whether the change in two assessment results was real or caused by random testing errors. It was considered that changed scores were real when the change value was greater than MCID [29]. The changed scores (T_0_-T_1_) of TIS, BBS and BI were 3.5, 4 and 10 points, respectively which all exceeded the MCID values, indicating that patients’ scores had really changed after 3-weeks treatment.

**Table 4:** Assessment results at T0 and T1 and MCID of TIS, BBS and BI. Data expressed as Median (Q1-Q3)

	**TIS (*n* = 60)**	**BBS (*n* = 60)**	**BI (*n* = 60)**
T_0_	13 (9.3–14)	10 (1–23)	45 (35–55)
T_1_	16 (14–19)	16.5 (4.3–32)	55 (45–65)
T_0_-T_1_	3.5 (2–4.8)	4 (2–8)	10 (5–15)
MCID	1.5	3.4	4.3

*TIS* Trunk Impairment Scale, *BBS* Berg balance scale, *BI* Barthel index, *T0* Time to assessment at admission, *T1* Time to assessment at discharge, *MCID* Minimal clinically important difference

Table 5 shows the improvements in respiratory muscle strength and pulmonary volume. MIP at T_1_ (38.1 ± 2.0) was significantly higher than at T_0_ (31.0 ± 1.8) (*P* < 0.001). MEP at T_1_ (35.4 ± 1.9) was significantly greater than at T_0_ (27.2 ± 1.6) (*P* < 0.001). FVC significantly increased from 65.9 ± 2.5 (T_0_) to 71.9 ± 2.5 (T_1_) (*P* < 0.001). FEV_1_ significantly promoted from 59.7 ± 2.7 (T_0_) to 68.7 ± 2.6 (T_1_) (*P* < 0.001). PEF significantly improved from 29.2 ± 1.8 (T_0_) to 36.8 ± 2.0 (T_1_) (*P* < 0.001). MMEF were 46.5 ± 2.9 (T_0_) and 57.5 ± 3.5 (T_1_), significantly difference(*P* < 0.001).

**Table 5:** TIS, BBS, BI, Pulmonary ventilatory function and respiratory muscle strength variation (Mean ± SD) after 3-week rehabilitation

	**T**_**0**_	**T**_**1**_	***P***
MIP (%)	31.0 ± 1.8	38.1 ± 2.0	< 0.001
MEP (%)	27.2 ± 1.6	35.4 ± 1.9	< 0.001
FVC (%)	65.9 ± 2.5	71.9 ± 2.5	< 0.001
FEV_1_ (%)	59.7 ± 2.7	68.7 ± 2.6	< 0.001
PEF (%)	29.2 ± 1.8	36.8 ± 2.0	< 0.001
MMEF (%)	46.5 ± 2.9	57.5 ± 3.5	< 0.001

*T*_*0*_ The time of admission, *T*_*1*_ The time after 3 weeks of treatment

**Fig. 4:**
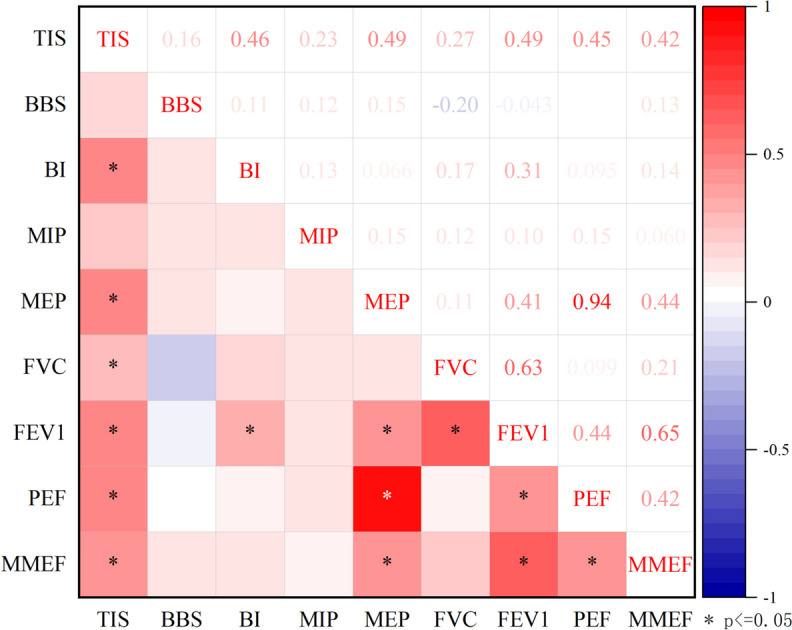
Spearman’s correlation between variations of motor function and variations of spirometric data

The results of Spearman’s correlation coefficient analysis are in Fig. 4. △ was used to represent the variation of the parameters. △TIS had significant positive correlations with △MEP (*r* = 0.49, *P* < 0.001), △FVC (*r* = 0.27,* P* = 0.034), △FEV_1_ (*r* = 0.49, *P* < 0.001), △PEF (*r* = 0.45, *P* < 0.001) and MMEF (*r* = 0.42, *P* = 0.001). △BI showed significant positive correlations with △FEV_1_ (*r* = 0.31,* P* = 0.016).

**Fig. 5:**
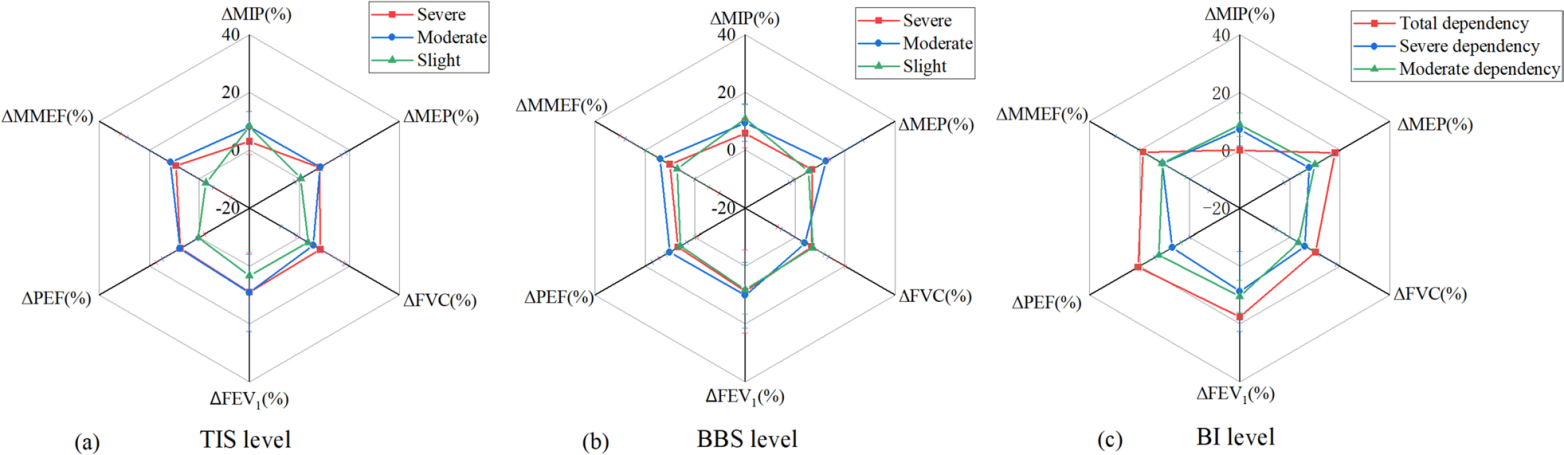
Variation of spirometric data at 3 levels of TIS (**a**), BBS (**b**) and BI (**c**) after 3 weeks rehabilitation

The results demonstrated that △MIP were 2.6(0–4.7), 6.4(4.5–11.1) and 8.3, with significant differences (*P* = 0.026), at severe, moderate, and slight TIS levels, respectively (Table 6, Fig. 5a). Less improvement of inspiratory pressure was found in the severe level of a trunk control disorder. The △MIP were 5 (2.5–8.5), 9 (4.8–12.6) and 9.7 (7.3–14.6), with a significant difference (*P* = 0.044), for severe, moderate, and mild BBS levels, respectively (Table 6, Fig. 5b). Respiratory parameters showed no differences in 3 different BI levels (Table 6, Fig. 5c).

**Table 6:** Variation (△) of Spirometric data at three BBS and BI levels after 3-week rehabilitation. Data expressed as Median (Q1-Q3)

	**Trunk Impairment Scale (TIS)**				**Berg Balance Scale (BBS)**				**Barthel Index (BI)**			
	**Severe (*N* = 10)**	**Moderate (*N* = 49)**	**Slight (*N* = 1)**	***p***	**Severe (*N* = 40)**	**Moderate (*N* = 16)**	**Slight (*N* = 4)**	***p***	**Total (*N* = 1)**	**Severe (*N* = 54)**	**Moderate (*N* = 5)**	***p***
△MIP (%)	2.6 (0–4.7)	6.4 (4.5–11.1)	8.3	0.026	5 (2.5–8.5)	9 (4.8–12.6)	9.7 (7.3–14.6)	0.044	0	6 (3.5–10.9)	11 (8.3–11)	0.204
△MEP (%)	5.8 (1.7–10.7)	6 (1.7–11.4)	0.5	0.392	5.2 (1.5–10.2)	10.6 (4.3–14.5)	3.6 (0.9–10.2)	0.065	18.1	5.5 (1.5–10.7)	10.9 (10.5–11)	0.234
△FVC (%)	9.7 (3.1–14.6)	6.9 (-0.5–12.6)	3.5	0.599	7.9 (1.8–13.2)	5.4 (-3.1–9.8)	6.1 (3.7–10.8)	0.475	10.3	7.6 (-0.5–13.1)	4.5 (3.5–6.3)	0.685
△FEV_1_ (%)	8.3 (0.7–19.0)	7.8 (2.2–18.0)	3.2	0.825	8 (1.3–18.3)	9.3 (3.8–18.3)	5.5 (2.3–14)	0.044	17.5	7.6 (1.8–19)	11.5 (6.9–13)	0.709
△PEF (%)	3.9 (1.3–11.3)	7.2 (2.2–11.8)	0.4	0.389	4.6 (1.3–11.3)	9.5 (4.9–14.3)	4.8 (2.2–9.9)	0.056	20.7	5.7 (1.7–10.9)	11.8 (7.8–20.5)	0.135
△MMEF (%)	4.6 (0.2–20.3)	10.2 (1.9–20.8)	-2.6	0.382	7.3 (1.1–21)	10.3 (6.8–20.6)	2.6 (-0.7–14.9)	0.236	18.7	7.5 (1.3–21.6)	10.5 (6.9–19.7)	0.82

## Discussion

This study analyzed variations in respiratory functions in patients with different motor disabilities and motor functions, and differences in these indicators at baseline and after 3 weeks treatment. Abnormal inspiratory muscle strength resulted in lower balance skills and activities of daily living, but not in trunk control. Regular training enhanced the strength of both inspiratory and expiratory muscles in stroke patients. Those with mild motor impairment demonstrated greater improvement in their inspiratory muscles strength after therapy.

### Correlations between motor and pulmonary function and respiratory muscles

TIS was used to evaluate the capacity of trunk muscles to maintain an upright posture and execute targeted movements during both static and dynamic postural modifications [30]. BBS assessed a patient’s ability actively to shift the center of gravity. BI was used to measure performance in activities of daily living. MIP and MEP revealed the strength of the maximum inspiratory and expiratory muscles. FVC and FEV_1_ were indicators that directly reflect the respiratory capacity, which depends on muscular strength [31, 32]. PEF and MMEF refer to the maximum expiratory flow rate and the average respiratory flow rate in the mid-section during forced exhalation, respectively. These parameters were primarily influenced by lung volume and expiratory muscle strength [33, 34].

It was found that TIS, BBS, and BI were positively correlated with MIP, MEP, FVC, FEV_1_, PEF, and MMEF in the present study. The results showed consistency with Fuglmyer’s study, indicating that respiratory dysfunction is prevalent in stroke patients with hemiplegia [2]. BBS and BI exhibited a moderate correlation with MIP in our study. The results also showed that FVC, FEV_1_, PEF and MMEF were correlated with respiratory muscle strength, especially the expiratory muscles. Further analysis of the variations in respiratory function among different levels of motor impairment were undertaken.

### Respiratory function analysis of different motor function levels

The study found notable differences in MIP, MEP and pulmonary function indicators at three levels of TIS, as well as BBS and BI. In contrast, there were significant differences in BBS and BI at two levels of MIP and 5 levels of FEV_1_. These results are consistent with the previous findings that BBS and BI were moderately correlated with MIP.

The decrease in TIS score after a stroke was primarily due to a reduction in selective trunk activities, which included the ability to perform unconstrained active movements of the trunk such as flexion, dorsiflexion, lateral flexion, and rotation [35]. Patients may exhibit abnormal trunk movement patterns, which require more energy for trunk activities [36] and can lead to a solidification pattern of the trunk, back extensor spasms, limited thoracic movement and diaphragm activity. These factors further inhibit the activity of the deep and superficial core muscles. TIS reflects the ability to move the torso flexibly and does not directly result in changes in respiratory muscle strength or lung volume [37]. Restricted trunk mobility indirectly resulted in a restrictive respiratory syndrome [38].

The lower the BBS and BI scores, then lower pulmonary function parameter values were recorded. A pervious study had also indicated that balance was independently associated with individual activities and participation [39]. This study found a strong correlation between BBS and BI. The analysis of the relationship between BI and respiratory function yielded a similar relationship between balance and respiratory function.

The inspiratory and expiratory muscles are the deep core muscles that directly affected a patient’s the ability to maintain trunk stability, rather than the selective control ability of the trunk. Therefore, there was no difference in MIP or MEP whether TIS was normal or not. However, patients with normal inspiratory muscles had higher BBS scores than those with abnormal activity, but the BBS scores did not differ between those with normal and abnormal MEP as well as for BI. The results indicated that inspiratory muscles were more involved in balance functions or daily activities than expiratory muscles, implying that the inspiratory muscles were more important for maintaining body balance and daily activities [40].

In brief, various motor functions had direct or indirect effects on respiratory function. Conversely, the inspiratory muscles had a direct impact on a patient’s balance and daily activities.

### Changes in motor and pulmonary function and respiratory muscle after 3 weeks rehabilitation

Bobath selective trunk postural control was an essential component of routine rehabilitation training, including turnover and bridge exercise training in the supine position, shifting the torso in the sitting and standing positions, and activating trunk muscles with ball activities. Our study found that after 3 weeks of routine rehabilitation without specific respiratory exercises, increased motor function also improved the strength of the respiratory muscles and pulmonary volume. Changes in TIS were moderately positively correlated with changes in MEP, FEV1, PEF, and FVC.

The results suggest that routine rehabilitation focusing on facilitating active and flexible trunk movement will significantly improve TIS, and will reflect enhanced control of trunk. Deep core muscles, such as the abdominal muscles, which are also the main exhalation muscles, were activated [41]. This activation explains the results, which found that changes in TIS were moderately correlated with changes in MEP. Of a correlation between TIS and MIP was not found, as also reported in the study by Jandt [42]. Zheng’s study demonstrated that routine rehabilitation improved diaphragm mobility but not its thickness [43], implying that routine training did not improve inspiratory muscle strength. Therefore, based on routine rehabilitation, it was necessary to improve the inspiratory muscle strength of patients with hemiplegia through multiple means, including increasing the thoracic range of motion, diaphragmatic mobilization, and muscle strength training.

Moreover, we also found that MIP improved in various ways in patients with different trunk control abilities. Patients with severe trunk control disorders showed less improvement. Possible reasons for this findings included: (1) in routine rehabilitation, patients received passive training supplemented by low-dose active training, which cannot effectively activate the diaphragm; (2) inspiratory training was often neglected due to poor patient cooperation; and (3) the therapist may neglect the active and passive training of the diaphragm in clinical treatment. Therefore, specific inspiratory muscle training should be included in the clinical rehabilitation process, especially for patients with severe trunk control disorders.

After 3 weeks treatment, we observed differences in the improvement of MIP among individuals with varying levels of balance disorders. Patients with higher balance abilities achieved a better degree of improvement in their core muscle groups, which was beneficial for the original role of the respiratory muscles [3].

The present study had some limitations, such as the small number of patients studied with certain impairment levels, which made it difficult to explain certain issues. Additionally, the 3-week treatment period was relatively short for stroke patients due to hospitalization requirements. Future studies will increase the treatment time to observe its impact on the respiratory muscles and pulmonary function.

## Conclusions

This research found that patients with hemiplegia exhibited suboptimal respiratory muscle strength and pulmonary function in the presence of more severe motor dysfunction. Impaired inspiratory muscle strength was found to be associated with a reduced balance ability and limitations in activities necessary for daily living, while trunk control remained unaffected. The functions of the inspiratory muscles, particularly the diaphragm, were crucial for both respiratory and motor functions. Routine rehabilitation enhanced exercise capacity and improved the activity of the expiratory muscles more than the inspiratory muscles. Therefore, in clinical settings, enhancing motor function may improve respiratory function. However, it is imperative for therapists to prioritize the activation of diaphragm function in critically ill patients.

## Data Availability

The datasets are available from the corresponding author on reasonable request.

## Abbreviations

TIS: Trunk Impairment Scale
BBS: The Berg Balance Scale
BI: The Barthel Index
MIP: Maximal inspiratory pressure
MEP: Maximal expiratory pressure
FVC: Forced vital capacity
FEV_1_: Forced expiratory volume in 1 s
PEF: Peak expiratory flow
MMEF: Maximal mid-expiratory flow
